# The utility of neonatal sequential organ failure assessment in mortality risk in all neonates with suspected late-onset infection

**DOI:** 10.1038/s41372-025-02304-2

**Published:** 2025-04-18

**Authors:** Faris N. Al Gharaibeh, Star Liu, James L. Wynn, Khyzer B. Aziz

**Affiliations:** 1 Division of Neonatology, Cincinnati Children’s Hospital Medical Center, Cincinnati, OH USA; 2 Department of Pediatrics, University of Cincinnati College of Medicine, Cincinnati, OH USA; 3 Department of General Internal Medicine, Biomedical Informatics and Data Science, Johns Hopkins University, Baltimore, MD USA; 4 Department of Pediatrics, University of Florida, Gainesville, FL USA; 5 Department of Pediatrics, Johns Hopkins University, Baltimore, MD USA

**Keywords:** Prognosis, Infectious diseases, Prognostic markers

## Abstract

**Objective:**

Assess the utility of the neonatal sequential organ failure assessment score (nSOFA) for evaluation-specific mortality discrimination in all late-onset infection (LOI) evaluations.

**Methods:**

Retrospective Cohort of all neonates who had an LOI evaluation from 2012 to 2023 in a single level IV Academic NICU in Florida, USA. The primary outcome was LOI-evaluation-specific mortality.

**Results:**

1481 neonates had 2916 LOI evaluations with a 3.8% mortality rate. The AUROC for the nSOFA score at evaluation was 0.76 (95% CI 0.71–0.81) and improved to 0.82 (95% CI 0.78–0.87) six hours after. nSOFA ≥2 within 6 h of the start of the LOI was 87% sensitive and 66% specific, with a 99% NPV for mortality, *p* < 0.0001.

**Conclusions:**

The nSOFA score had good to excellent mortality discrimination at the LOI evaluation level. These results solidify the utility of the nSOFA score as the foundation for a consensus definition of neonatal sepsis.

## Introduction

Sepsis continues to be a significant cause of death and morbidity in neonates [1], especially among those born preterm [2, 3]. Consensus definitions for sepsis in adult and pediatric patients [4, 5] have facilitated research and the development of surviving sepsis guidelines for over two decades [6, 7]. The recent pediatric consensus definition appropriately and specifically excluded preterm neonates and neonates hospitalized immediately after birth [5], mainly due to their unique developmental and transitional physiology and pathology. Thus, this unique subset of patients is the only remaining population without a supportive consensus definition for sepsis. This deficit severely hinders much-needed research on these patients, including the development of diagnostic and prognostic tests for those who are heavily affected by sepsis [8].

The neonatal sequential organ failure assessment (nSOFA) is an objective multi-organ dysfunction scoring system that includes respiratory, cardiovascular, and coagulation systems [9]. The nSOFA is not a *diagnostic* test but rather a validated illness severity score that operationalizes life-threatening organ dysfunction among neonates cared for in the NICU, specifically preterm neonates and neonates hospitalized immediately after birth. In multiple multicenter studies of the NICU patient population, the nSOFA demonstrated good to excellent discrimination for all-cause mortality (*n* = 20152) [10], as well as specifically for patients with early-onset infection (≤72 h after birth; *n* = 104) [11], late-onset infection (>72 h after birth; *n* = 713) [12], and necrotizing enterocolitis (NEC) (*n* = 259) [13]. The nSOFA-focused studies of early/late infection and NEC included only patients with retrospectively confirmed disease, which is very unlikely to be known at the time of empiric treatment and enrollment in a prospective observational or therapeutic clinical investigation.

It is unknown if the nSOFA retains the utility of predicting mortality among patients with suspected infection when all evaluations of suspected infection are examined. If the nSOFA demonstrated utility to predict evaluation-level mortality early during empiric treatment for suspected infection, this would represent a valuable step towards developing a consensus definition in this population and provide a useful tool to classify patients enrolled in clinical investigations with neonatal sepsis. Here, we aimed to test the hypothesis that the nSOFA has good mortality discrimination early after late-onset infection (LOI) evaluation in all NICU patients.

## Methods

We conducted a single-center observational retrospective cohort study of all infants admitted to the University of Florida (UF) Health Shand’s Children’s Hospital NICU from 1/1/2012 to 4/1/2023 with an LOI evaluation. Neonates of all gestational ages and birth weights were included. The University of Florida Institutional Review Board approved the study and granted a waiver of consent.

We defined an LOI evaluation as a new, broad-spectrum parenteral antibiotic administration that occurred more than 72 h after birth without antibiotic exposure in the prior 48 h. If a neonate experienced multiple evaluations during hospitalization, *each evaluation was included and analyzed as an individual event*. All neonates were included regardless of the duration of antibiotic therapy, birth weight, sex at birth, presence of congenital anomalies, and code status to encompass all possible events that may occur in the clinical setting.

Using electronic health record data, nSOFA scores were calculated at two discrete time points (at evaluation; time 0 (T0) and 6 h after evaluation (T6)) as previously described [12]. The nSOFA includes respiratory, cardiovascular, and hematologic dysfunction measures applicable to the NICU population. The score components were calculated based on the most recent available data (intubation status, inspired oxygen fraction, oxygen saturation, vasopressor or systemic steroid use, and platelet count) in relation to T0 and T6. A last occurrence copied forward approach was applied to platelet values only. All other nSOFA components were available hourly.

The primary outcome was LOI-evaluation-specific mortality, defined as death that occurred while the infant was receiving antibiotic therapy for a given LOI evaluation.

Categorical variables were compared using the Chi-square test. Non-parametric continuous variables were compared using the Mann-Whitney test. The change in the nSOFA score between T0 and T6 was done using the Wilcoxon test. Discrimination ability was determined by measuring the area under the receiver operating characteristics (AUROC) curve. The Youden’s J statistic was used to determine the best nSOFA cutoff for mortality. Test characteristics’ 95% confidence intervals were calculated using the Wilson method. Statistical analyses were conducted using GraphPad Prism (GraphPad Software, San Diego, CA, USA). A two-sided *p* < 0.05 was determined significant.

## Results

### Cohort characteristics

1481 neonates, with an overall mortality rate of 126/1481 (8.5%), were included during the study period who had 2916 LOI evaluations. Of those evaluations, 328 (11%) were associated with bacterial isolation from the blood or the cerebrospinal fluids, and 110 (3.8%) ended in mortality. The median time of death was 5 [IQR 2-12] days. Compared to survivors (*n* = 1371), neonates who experienced mortality (*n* = 110) were born at an earlier gestational age [32 [interquartile range (IQR) 26–36] weeks vs. 33 [IQR 27–38] weeks, p 0.047], weighed less at birth [1560 [IQR 746–2559] grams vs. 1780 [IQR 890–2892] grams, p 0.024], and were more likely to have major congenital anomalies [44/110 (40%) vs. 382/1371 (28%), p 0.009]. The total number of evaluations experienced by an infant did not differ between survivors and non-survivors (Table 1).

**Table 1:** Characteristics of neonates according to primary outcome.

	Mortality (*n* = 110)	Survival (*n* = 1371)	*p* value
Gestational Age (weeks)^a^	32 [26–36]	33 [27–38]	0.047^b^
Birthweight (grams)^a^	1560 [746–2559]	1780 [890–2892]	0.024^b^
Female	58 (53%)	612 (45%)	0.101^c^
C-section	68 (62%)	781 (57%)	0.367^c^
Parent-declared race: Black	40 (36%)	449 (33%)	
White	54 (49%)	735 (54%)	0.652^c^
Other	16 (15%)	187 (13%)	
Hispanic Ethnicity	8 (7%)	123 (9%)	0.546^c^
Major Congenital Anomalies^d^	44 (40%)	382 (28%)	0.009^c^
Total Number of Evaluations^a^	1 [1–3]	1 [1–2]	0.158^b^

^a^Data displayed as the median and 25th–75th interquartile range.

^b^Calculated using the Mann–Whitney test.

^c^Calculated using the Chi-Square test.

^d^Congenital heart disease other than ASD, VSD or PDA, congenital diaphragmatic hernia, anomalies of the gastrointestinal tract, anomalies of the central nervous system, and anomalies of the kidney and the urinary tract (CAKUT).

### nSOFA score distribution and LOI-specific mortality

**Fig. 1:**
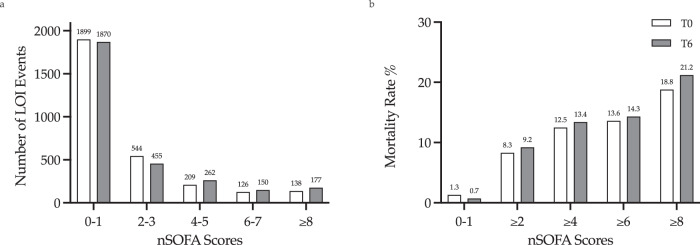
Distribution of nSOFA scores in LOI evaluations and mortality. **a** The nSOFA scores at T0 (white) and T6 (gray) in LOI evaluations. The number of evaluations with each nSOFA score category is shown above the bar. **b** Mortality rate according to nSOFA score at T0 and T6.

**Fig. 2:**
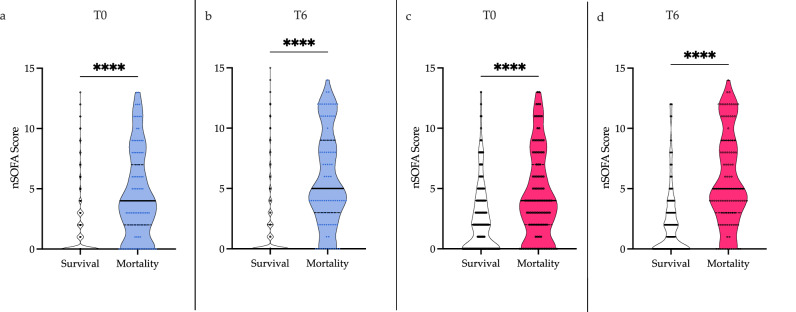
nSOFA scores in LOI evaluations. **a**, **b** nSOFA scores according to mortality (dark blue) and survival (white) at T0 and T6 in all evaluations (*n* = 2916). **c**, **d** nSOFA scores according to mortality (pink) and survival (white) in neonates who ultimately died from their last LOI (*n* = 292). ****p < 0.0001 by the Mann–Whitney test.

More than 60% of the events had nSOFA scores of 0–1 at T0 and T6. The distribution of scores is summarized in Fig. 1a. Evaluations that ended in mortality had higher nSOFA scores at T0 compared to those ending in survival, 4 [IQR 2–7] vs. 0 [IQR 0–2] respectively, *p* < 0.0001 (Fig. 2a). At the T6 time point, evaluations that ended in mortality had a median score of 5 [IQR 3–9] vs. 0 [IQR 0–2] in those ending in survival, *p* < 0.0001 (Fig. 2b). Mortality rates and nSOFA scores at T0 and T6 were positively correlated (Fig. 1b). Evaluations in neonates with scores ≤1 had low mortality (0.7–1.3%), while those with scores ≥2 had higher mortality (8.3–9.2%). Furthermore, evaluations ending in survival had minimal change in their nSOFA scores between T0 and T6 with a mean change of 0.14 (95% CI 0.09–0.19), while evaluations ending in mortality had a mean change of 1.3 (95% CI 0.8–1.82), *p* < 0.0001.

### nSOFA score and LOI-specific mortality

The AUROC for the nSOFA score at T0 was 0.76 (95% confidence interval (CI) 0.71–0.81), *p* < 0.0001, which improved at T6 to 0.82 (95% CI 0.78–0.87), *p* < 0.0001. An nSOFA score of 2 or greater (Youden’s J statistic) was used as a cutoff for mortality testing (Table 2).

**Table 2:** Test Characteristics of nSOFA ≥ 2 in Mortality Prediction in All LOI Evaluations (*n* = 2916).

	Zero Hour	+6 h
Sensitivity	76% (67–83%)	87% (80–93%)
Specificity	67% (65–69%)	66% (64–68%)
NPV	99% (98–99%)	99% (99–100%)
PPV	8% (7–10%)	9% (8–11%)
+LR	2.3	2.6
−LR	0.36	0.20
AUC	0.76 (0.71–0.81)	0.82 (0.78–0.86)

Values between parenthesis represent the 95% CI.

*NPV* negative predictive value, *PPV* positive predictive value, *LR* Likelihood ratio, *AUC* Area under the curve for the ROC curve.

We also performed a restricted analysis that included only LOI evaluations in neonates who ultimately died with their last LOI. Among the 110 non-survivors, there were 292 LOI evaluations of which 182 LOI evaluations ended in survival and 110 (38%) resulted in death. nSOFA scores remained significantly different at both T0 and T6 between evaluations ending in mortality and those ending in survival (T0: 4 [IQR 2–7] vs. 2 [IQR 0–4] (Fig. 2c) and T6: 5 [IQR 3–9] vs. 1 [IQR 0–3] (Fig. 2d) respectively, *p* < 0.0001). In this sub-group of evaluations, the AUROC for the nSOFA at T0 was 0.65 (95% CI 0.59–0.72), *p* < 0.0001, and increased at T6 to 0.77 (95% CI 0.72–0.83), *p* < 0.0001. The test characteristics of nSOFA ≥2 in this group are summarized in Table 3.

**Table 3:** Test Characteristics of nSOFA ≥ 2 in Mortality Prediction in LOI Evaluations (*n* = 292) for Neonates Who Ultimately Died.

	Zero Hour		+6 h
Sensitivity	77% (68–83%)		87% (80–92%)
Specificity	43% (36–51%)		41% (34–48%)
NPV	75% (66–83%)		84% (75–90%)
PPV	45% (38–52%)		47% (41–54%)
+LR	1.3		1.5
−LR	0.53		0.32
AUC	0.65 (0.59–0.72)		0.77 (0.72–0.83)

Values between parenthesis represent the 95% CI.

*NPV* negative predictive value, *PPV* positive predictive value, *LR* Likelihood ratio, *AUC* Area under the curve for the ROC curve.

### nSOFA score to discriminate for LOI-specific mortality by birth weight

Since most reported infections occur in very low birth weight (VLBW, <1500 g) neonates, we conducted separate analyses restricted to this population. In VLBWs, there were 1330 LOI evaluations, with 52 that ended in mortality (3.9%). In this group, the AUROC for the nSOFA score to discriminate mortality at T0 was 0.79 (95% CI 0.72–0.86), *p* < 0.0001, which increased at T6 to 0.87 (95% CI 0.82–0.92), *p* < 0.0001. Table 4 summarizes the test characteristics of nSOFA ≥ 2 in VLBW neonates. Neonates with birthweight >1500 g had 1586 LOIs with 58 ending mortality (3.7%). In this group, the AUROC for mortality discrimination at T0 and T6 were 0.73 (95% CI 0.65–0.80) and 0.77 (95% CI 0.71–0.84), respectively, *p* < 0.0001.

**Table 4:** Test Characteristics of nSOFA ≥ 2 in Mortality Prediction in LOI Evaluations (*n* = 1331) For VLBW Neonates.

	Zero Hour	+6 h
Sensitivity	77% (64–86%)	90% (79–96%)
Specificity	69% (67–72%)	69% (66–71%)
NPV	99% (98–99%)	99% (99–100%)
PPV	9% (7–12%)	11% (8–14%)
+LR	2.5	2.9
−LR	0.36	0.14
AUC	0.79 (0.72–0.86)	0.82 (0.78–0.86)

Values between parenthesis represent the 95% CI.

*NPV* negative predictive value, *PPV* positive predictive value, *LR* Likelihood ratio, *AUC* Area under the curve for the ROC curve.

## Discussion

In this study, where every late-onset infection evaluation was included over an 11-year period, the nSOFA demonstrated good to very good discrimination for the outcome of mortality associated with a given evaluation in all NICU patients and very good to excellent discrimination in VLBWs. Early discrimination for evaluation-level mortality risk remained good even when analyses were restricted to evaluations in patients who ultimately died. The nSOFA identified life-threatening organ dysfunction at the evaluation level, which adds to the existing evidence [12, 14, 15] that supports the use of the nSOFA as a starting point for a consensus definition of neonatal sepsis.

Consensus sepsis definitions in children and adults require life-threatening organ dysfunction. To objectively quantify life-threatening organ dysfunction, the sequential organ failure assessment (SOFA) score is used for adults, while the Phoenix Sepsis Score is used for pediatric patients [4, 5]. The SOFA score includes assessments for organ dysfunction in six systems (respiratory, cardiovascular, hematologic, hepatic, renal, and neurologic) [16]. The Phoenix sepsis score includes assessments for organ dysfunction in four systems (respiratory, cardiovascular, hematologic, and neurologic) [17]. Like the Phoenix Score, the nSOFA includes assessments for organ dysfunction in three systems (respiratory, cardiovascular, and hematologic) and has been tested and validated for the NICU population, including term and preterm infants, in multiple cohorts. Multiple multicenter studies have demonstrated the utility of the nSOFA to accurately discriminate for mortality in NICU patients with confirmed early-onset [11] and late-onset bacteremia [12], necrotizing enterocolitis [13], and among the NICU population for all-cause mortality [10]. The current study showed the nSOFA had strong utility for mortality among all NICU patients (no exclusions) and included all evaluations for late-onset infection. Taken together, the nSOFA, like the SOFA in adults and the Phoenix score in children, has demonstrated utility in quantifying life-threatening organ dysfunction among those with suspected infection in the NICU population and could serve as a starting point for a consensus definition of neonatal sepsis.

These data suggest the nSOFA is likely to improve early patient classification and facilitate prognostic enrichment of NICU patients enrolled in future interventional clinical studies focused on the outcome of sepsis mortality. In this study, most neonates (65%) evaluated for LOIs had an nSOFA score of ≤1 at evaluation and at 6 h after, with a very low mortality rate (≤1%). Detailed patient classification is required for precision medicine approaches in any population [18]. A means to reduce patient exposure to potential deleterious side effects of an intervention when there is a very low risk of mortality would be an important contribution to neonatology clinical studies. Using the nSOFA could decrease group heterogeneity, facilitate equitable study arm enrollment, and reduce the number of neonates needed to achieve adequate power in clinical trials through better patient classification. Beyond the outcome of mortality, the nSOFA has demonstrated utility in identifying the risk of BPD, ROP, and NDI among neonates with sepsis [19, 20].

This study has limitations. This is a single-center study, and the practices unique to the center could influence the generalizability of the results. To mitigate the potential impact of bias, we included every LOI evaluation from over 11 years that occurred in every patient cared for in a level IV academic referral NICU, regardless of the duration of antibiotic therapy, presence of congenital anomalies, and the infant’s code status. We could not confirm that death was the direct result of LOI. However, we defined LOI mortality as death occurring while receiving antibiotics prescribed at the clinician’s discretion for the LOI evaluation. We could not determine the suspected infection source that prompted the LOI evaluation and thus could not perform primary site of suspected LOI-specific analyses.

## Conclusions

The nSOFA score had good to excellent mortality discrimination at the LOI evaluation level. These results further solidify the utility of the nSOFA score as a stratification tool for LOI evaluations and as the foundation for a consensus definition of sepsis in neonates.

## Data Availability

The results summarize the data used for this study. For further inquiries, contact the corresponding author.
